# Whole-Genome Resequencing Analysis of Athletic Traits in Grassland-Thoroughbred

**DOI:** 10.3390/ani15152323

**Published:** 2025-08-07

**Authors:** Wenqi Ding, Wendian Gong, Tugeqin Bou, Lin Shi, Yanan Lin, Xiaoyuan Shi, Zheng Li, Huize Wu, Manglai Dugarjaviin, Dongyi Bai

**Affiliations:** 1 Key Laboratory of Equus Germplasm Innovation (Co-Construction by Ministry and Province), Ministry of Agriculture and Rural Affairs, Hohhot 010018, China; dingwenqi0331@gmail.com (W.D.); gongwendian1996@outlook.com (W.G.); tvgqin@gmail.com (T.B.); 19832607527@163.com (L.S.); linyanan@emails.imau.edu.cn (Y.L.); xiaoyuans2021@163.com (X.S.); lzheng0511@sina.com (Z.L.); whz020419@163.com (H.W.); dmanglai@163.com (M.D.); 2 Inner Mongolia Key Laboratory of Equine Science Research and Technology Innovation, Inner Mongolia Agricultural University, Hohhot 010018, China; 3 Equus Research Center, College of Animal Science, Inner Mongolia Agricultural University, Hohhot 010018, China

**Keywords:** Grassland-Thoroughbred, hybrid offspring, racehorses, whole-genome, athletic selection traits

## Abstract

Speed is not only the primary objective of racehorse breeding but also a crucial indicator for evaluating racehorse performance. This study investigates a newly developed racehorse breed in China. Through whole-genome resequencing, we selected 60 offspring obtained from the crossbreeding of Thoroughbred horses and Xilingol horses for this study. This breed is tentatively named “Grassland-Thoroughbred”, and the samples were divided into two groups based on racing ability: 30 racehorses and 30 non-racehorses. Based on whole-genome sequencing data, the study achieved an average sequencing depth of 25.63×. The analysis revealed strong selection pressure on chromosomes (Chr) 1 and 3. Selection signals were detected using methods such as the nucleotide diversity ratio (π ratio), integrated haplotype score (iHS), fixation index (Fst), and cross-population extended haplotype homozygosity (XP-EHH). Regions ranked in the top 5% by at least three methods were designated as candidate regions. This approach detected 215 candidate genes. Additionally, the Fst method was employed to detect Indels, and the top 1% regions detected were considered candidate regions, covering 661 candidate genes. Functional enrichment analysis of the candidate genes suggests that pathways related to immune regulation, neural signal transmission, muscle contraction, and energy metabolism may significantly influence differences in performance. Among these identified genes, *PPARGC1A*, *FOXO1*, *SGCD*, *FOXP2*, *PRKG1*, *SLC25A15*, *CKMT2*, and *TRAP1* play crucial roles in muscle function, metabolism, sensory perception, and neurobiology, indicating their key significance in shaping racehorse phenotypes. This study not only enhances understanding of the molecular mechanisms underlying racehorse speed but also provides essential theoretical and practical references for the molecular breeding of Grassland-Thoroughbreds.

## Simple Summary

Enhancing speed remains the primary objective in racehorse breeding programs. In this study, whole-genome resequencing was conducted on 30 racing-type and 30 non-racing-type Grassland-Thoroughbreds, with an average sequencing depth of 25.63×. Selection signals were detected using multiple methods, including π ratio, iHS, Fst, and XP-EHH, revealing strong selective pressure on chromosomes 1 and 3 and identifying a total of 215 candidate genes. Additionally, Fst analysis of Indels yielded 661 more candidate genes. Functional enrichment analysis indicated that pathways related to immune regulation, neural signal transmission, muscle contraction, and energy metabolism play crucial roles in differences in athletic performance. Among the identified genes, *PPARGC1A*, *FOXO1*, *SGCD*, *FOXP2*, *PRKG1*, *SLC25A15*, *CKMT2*, and *TRAP1* are closely associated with muscle function, metabolism, and neural regulation, suggesting their significant roles in shaping racehorse traits.

## 1. Introduction

Horses occupy a distinctive role in modern society, having transitioned significantly from their historical function as traditional working animals to their current roles in sports and recreation. Among contemporary equestrian activities, horse racing stands out as a globally popular sport, attracting widespread attention and participation [1]. Horse racing, typically involving jockeys competing on designated tracks, represents a major industry in at least 71 countries, with approximately 500,000 racehorses actively competing and offering over EUR 3.3 billion in prize money globally [2]. Internationally renowned events such as the Melbourne Cup, Kentucky Derby, and Japan Cup are highly celebrated [3,4,5]. Additionally, global horse racing betting generates annual revenues exceeding 115 billion EUR, making substantial contributions to local economic development, job creation, and the enrichment of recreational activities [5]. To achieve outstanding performance in specific types of competitions, various horse breeds have been gradually developed through selective breeding to meet the evolving demands of racing and athletic performance.

After more than 200 years of rigorous selective breeding, Thoroughbreds have become the most valuable and widely regarded elite racehorse breed globally. They are renowned for their exceptional athletic performance. In their early development, the enthusiasm for horse racing among British aristocrats significantly contributed to the refinement of this breed [6]. Thoroughbreds are celebrated for their outstanding athletic phenotype, excelling particularly in speed-based races due to their finely honed genetic and physical traits [7]. The Mongolian horse is one of the oldest existing horse breeds, renowned for its exceptional endurance, ability to thrive on coarse feed, and resistance to diseases [8]. As an important indigenous horse breed resource in China, Mongolian horses are frequently utilized as foundation stock for horse improvement programs. Among these, the Xilingol horse is a new breed developed through 35 years of targeted breeding, with the Mongolian horse serving as the maternal breed [9]. Since 1995, Thoroughbreds have been incorporated into the Xilingol horse breeding program for hybrid vigor. After nearly 30 years of meticulous breeding, significant progress has been achieved. The resulting offspring have been provisionally named the “Grassland-Thoroughbred” and are colloquially referred to as “Sunflower Horses” due to the distinctive sunflower-shaped markings on their hindquarters. As the genetic improvement of horses continues to advance, research is expanding from traditional traits such as body type and speed to genomic-level exploration. Genomic variations are increasingly recognized as crucial factors determining horses’ environmental adaptability and athletic ability.

In recent years, with the advent of genome resequencing technology, it has become possible to identify selection traits across the entire genome [10]. This innovative sequencing approach enables the detection of complete genomic information in individuals, leading to significant advancements in the study of racehorse performance. Racing ability is a complex trait determined by the interaction of multiple genes. For instance, the *MYPN* gene, which encodes myopalladin and is mainly expressed in skeletal and cardiac muscle, plays a crucial role in regulating actin organization and has undergone positive selection in horses [11,12]. The athletic potential of racehorses depends not only on favorable environmental conditions but also on optimal DNA variation combinations at loci that significantly influence athletic ability. A genome-wide SNP association study revealed that a sequence variation (g.66493737C>T) in the *MSTN* (myostatin) gene is the most effective indicator for predicting the optimal race distance for Thoroughbreds [13]. Additionally, Studies have also found that young Spanish trotters carrying the GG genotype of the *PDK4* gene and the AA genotype of the *DMRT3* gene perform exceptionally well in short-distance races, while horses with the AA genotype of *DMRT3* and the CC genotype of *CKM* excel in long-distance races. In adult horses, the AA genotype of *DMRT3* can enhance both speed and endurance [14]. SNPs and haplotypes in the *EDN3* gene region are significantly associated with racing performance. The T-allele is more frequent in racing breeds, while the CC genotype predominates in smaller horses and draft horses [15]. These findings indicate that genome resequencing is a comprehensive and reliable method for exploring important regions and functional genes associated with athletic traits. It provides a robust theoretical foundation and technical support for genetic improvement of racehorse performance.

This study systematically analyses the genetic variation between the racehorse and non-racehorse populations of Grassland-Thoroughbreds, with a particular focus on identifying SNP loci significantly associated with athletic performance. The findings offer valuable references for the genetic improvement and scientifically informed breeding of racehorse breeds. By optimizing breeding strategies, it is possible not only to significantly enhance the athletic performance of racehorses but also to promote the high-quality development of China’s horse racing industry. Additionally, this research contributes to strengthening the genetic diversity of Chinese horse breeds, contributing to the protection and sustainable utilization of equine resources.

## 2. Materials and Methods

### 2.1. Sample Collection

The Grassland-Thoroughbreds used in this study are offspring derived from multiple generations of crossbreeding between Xilingol horses and Thoroughbreds. These samples were obtained from Inner Mongolia Grassland-Thoroughbred Breeding Company Limited, which maintains complete pedigree records and breeding lineage information for the hybrid horses (Supplementary Figure S1). Based on the pedigree data, individuals with close genetic relationships were excluded to reduce genetic relatedness among samples. The experimental subjects consisted of two groups of Grassland-Thoroughbreds: a non-racing group (NR group, n = 30), whose members had no professional training or competition experience; and a racing group (RH group, n = 30), composed of individuals with consistent competitive performance. The performance of the RH group horses was evaluated through multiple regional competitions, and high-level racehorses achieving excellent rankings in multiple races were ultimately selected (Supplementary Table S1). Blood samples were collected from the jugular vein of each horse by professional technicians and placed into anticoagulant tubes containing EDTA. The samples were then aliquoted, rapidly frozen in liquid nitrogen, and stored at −80 °C to ensure sample integrity and stability for subsequent experiments.

### 2.2. Library Construction and Sequencing

Genomic DNA was first extracted from all blood samples using the CTAB method and subsequently used for DNA library preparation [16]. Sequencing libraries were then prepared following the manufacturer’s instructions using the NEBNext^®^ Ultra™ DNA Library Prep Kit for Illumina (NEB, San Diego, CA, USA), with unique index adapters ligated to each sample [17]. The purified libraries were assessed for quality and fragment size distribution using the Agilent 5400 system (Agilent, Santa Clara, CA, USA) to ensure they met the sequencing criteria. Libraries that passed quality control were subjected to paired-end sequencing (PE150) on the Illumina HiSeq 4000 platform (Novogene Co., Ltd., Beijing, China), generating high-quality sequencing data.

### 2.3. Data Quality Control

Firstly, the raw sequencing data were subjected to quality control using fastp (version 0.20.0) to ensure reliable reads for subsequent analyses. The quality control criteria included (1) removing reads with ≥5% unidentified nucleotides (N); (2) removing reads with >20% bases having a Phred quality score less than 15; (3) removing reads with adapter sequences, allowing ≤3% mismatches; and (4) removing reads shorter than 15 bases [18]. After cleaning, the processed data were evaluated using the FastQC (version 0.11.5) tool to verify their quality and suitability for subsequent analyses [19].

### 2.4. Variant Detection

The clean data were initially aligned to the horse reference genome (https://www.ncbi.nlm.nih.gov/datasets/genome/GCF_002863925.1/, accessed on 27 November 2020) using BWA (version 0.7.15) [20]. The resulting alignment files were converted and sorted using Samtools (version 1.3). The Picard tool (https://github.com/broadinstitute/picard, accessed on 25 August 2021) was used with the Picard MarkDuplicates command, setting REMOVE_DUPLICATES = true to exclude potential PCR duplicates. SNP calling was performed on the recalibrated BAM files using GATK’s HaplotypeCaller (version 4.0.3.0) [21], followed by merging with CombineGVCFs and obtaining the raw SNPs and raw Indels using the SelectVariants module. SNP files were filtered using the VariantFiltration parameter with the following conditions: QUAL < 30.0, QualByDepth (QD) < 1.5, RMS Mapping Quality (MQ) ≥ 4, Depth of Coverage (DP) < 5. After applying these filters, a VCF file containing high-quality SNPs was generated. Samtools flagstat was used to obtain alignment statistics for each sample. VCFtools was used for further filtering. SNPs missing in more than 80% of samples were excluded (--max-missing 0.8). Variants with a minor allele frequency below 0.05 (--maf 0.05) and only biallelic variants (--min-alleles 2 --max-alleles 2) were included. Retain sites with a QUAL score ≥ 30. SNPs not mapped to any autosomes or located on sex chromosomes (Chr) were excluded to ensure data accuracy and consistency.

To compare Indels between racing and non-racing horses and identify unique mutations, the raw Indels were obtained using the SelectVariants module. Quality control conditions for Indels were defined as follows: QD < 2.0, QUAL < 30.0, FS > 200.0, ReadPosRankSum < −20.0.

### 2.5. Principal Component Analysis

Based on the identified SNP data, PCA was conducted using PLINK (version 1.9) software to investigate the genetic structure of the racing and non-racing horse populations. The distribution of the first two principal components (PCs) was visualized using the ggplot2 package in R (version 4.4.1). The PIHAT (Proportion of Identical by Descent) values were estimated using the --genome command in PLINK software to calculate the homozygosity proportion between horses. Closely related individuals were removed (PIHAT value > 0.3).

### 2.6. Whole-Genome Selection Signal Analysis

Combining multiple methods of analysis can enhance the accuracy and sensitivity of genetic selection signals, partially addressing the limitations of small sample sizes. When multiple analysis methods indicate the same genetic variation signals, the authenticity of these signals can be confirmed with greater confidence [22,23]. Based on different principles, this study employed four selection signal detection methods to screen and analyze target regions and candidate genes.

Due to the susceptibility of single-point SNP scanning methods to interference from factors such as genetic drift, we employed a sliding window calculation strategy to enhance sensitivity to target signals and reduce the probability of false positives [24]. To detect genomic regions under positive selection between racing and non-racing horses, four test methods were used to analyze selection features. First, Fst (Fixation index) is an indicator of genetic differentiation between two populations and is widely applied to identify selection signals across the genome [25,26]. Second, the π ratio assesses selective pressure in genomic regions by comparing the nucleotide diversity (π) ratio between racing and non-racing populations to scan the genome for positively selected regions. Fst and π ratio values were computed using VCFtools (version 0.1.15) [27], with parameters set as follows: -fst-window-size (--window-pi) 100,000 and -fst-window-step (--window-pi-step) 10,000, followed by the calculation of the π ratio [28]. In both analyses, the top 5% of sites were extracted as candidate SNPs. Combining Fst and π ratio screenings allows for the identification of stronger selection signals and the further targeting of candidate genes.

Additionally, the Integrated Haplotype Score (iHS) and Cross-population Extended Haplotype Homozygosity (XP-EHH) were employed to identify positive selection signals within populations through haplotype structure analysis and are widely applied in livestock selection studies [29,30]. Under selection, the frequency of preferred alleles increases rapidly, forming long haplotype regions with low polymorphism, which are significantly distinct from regions with high polymorphism and low linkage disequilibrium in non-selected areas [31]. In this study, BEAGLE (version 5.1) was used with default settings to impute missing alleles for all individuals of Grassland-Thoroughbred and to infer haplotype phases [32]. The selscan (version 1.2.0) tool was employed to analyze selection signals by calculating the iHS (selscan --ihs) and XP-EHH (selscan --xpehh) values between two populations. Additionally, the norm module of selscan was further employed to standardize iHS (norm --ihs --bp-win --winsize 100000) and XP-EHH (norm --xpehh --bp-win --winsize 100000) scores. This study further calculated the average |iHS| and XP-EHH scores for non-overlapping windows of 100 kb across the entire genome of autosomal chromosomes [33,34]. We adopted the top 5% regions as the screening criterion, following a common empirical approach in selection signal studies.

### 2.7. Detection and Annotation of Candidate Genes

In this study, genome data were analyzed using the Fst, π ratio, iHS, and XP-EHH methods; SNPs falling within the top 5% of each selection metric were designated as “outlier loci”. Candidate regions associated with racehorse selection were defined as those identified by at least three of the four methods. Subsequently, genes containing these outlier loci were identified based on the annotation information of the EquCab 3.0 reference genome. Finally, a Venn diagram was constructed to visualize the overlap among the candidate gene sets identified by the Fst, π ratio, iHS, and XP-EHH methods.

### 2.8. Functional Enrichment Analysis

Gene Ontology (GO) and Kyoto Encyclopedia of Genes and Genomes (KEGG) enrichment analyses provide critical insights into the regulatory mechanisms of genes associated with traits like equine athletic performance, offering valuable directions for future research. To elucidate gene functions and their regulatory mechanisms, we performed functional enrichment analyses. Using the online tool DAVID (https://david.ncifcrf.gov/, accessed on 12 January 2025), we conducted functional enrichment analyses for GO and KEGG. To obtain more meaningful enrichment information, only annotations and pathways with a *p* < 0.05 were retained [35]. To better understand the functional dynamics among the candidate genes, a protein–protein interaction (PPI) network was constructed using the STRING database with Equus caballus as the reference species. The interaction data were imported into Cytoscape (version 3.7.1) for network visualization and further analysis.

## 3. Results

### 3.1. Sequencing and Detection of SNPs and Indels

**Figure 1:**
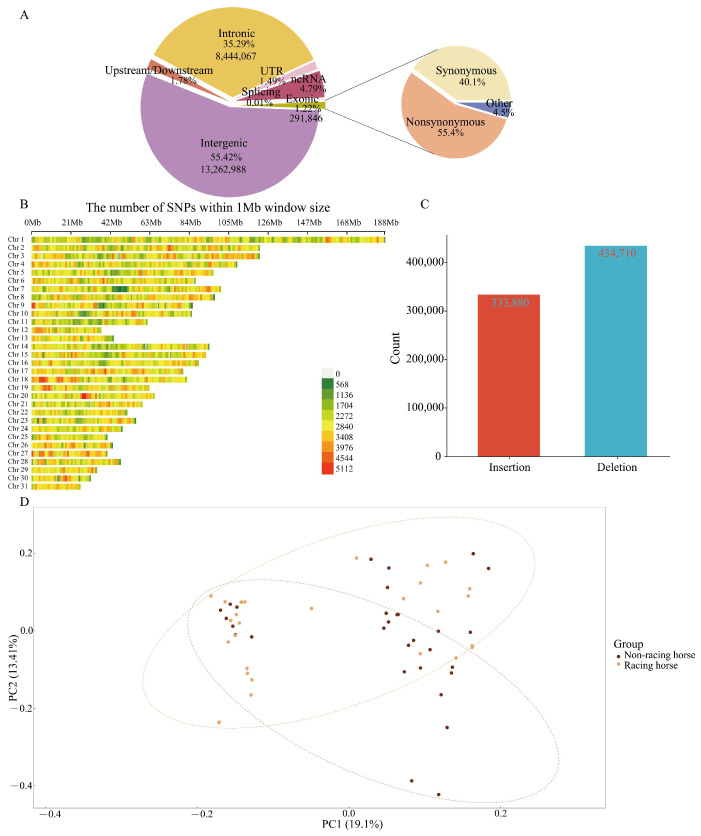
The quantity and distribution of SNPs, Indels, and population structure: (**A**) Annotation of genome-wide SNPs according to ANNOVAR. (**B**) The SNP density across the whole genome was estimated in each 1 Mb genome block. (**C**) Statistical analysis of insertion and deletion types of Indels. (**D**) Principal component analysis between racing and non-racing horses.

This study conducted high-depth genome re-sequencing on 60 horses, with an average individual genome sequencing depth of 25.63X. Alignment to the reference genome achieved 99.19% coverage (Supplementary Table S2). A total of 23,929,853 SNPs were identified (Figure 1A), the majority of which were located in intergenic regions and intronic regions, with relatively few in exonic regions. Analysis revealed 161,824 missense mutations and 116,897 synonymous mutations in protein-coding genes. The quality of the SNPs was assessed by calculating the transition-to-transversion ratio (Ti/Tv), which yielded a Ti/Tv ratio of 2.06 (Supplementary Table S3). Following stringent filtering, a total of 6,361,002 high-quality SNPs were retained. Genome-wide distribution analysis showed that SNP density was highest on Chr 1 and 3, and lowest on Chr 31 (Figure 1B). Additionally, this study identified 768,590 Indels, with the number of deletions (n = 434,710) slightly higher than insertions (n = 333,880) (Figure 1C). These Indels were distributed across the entire genome but exhibited uneven distribution, with the highest number of Indels located on Chr 1 (n = 55,984). Functional annotation using the ANNOVAR (https://annovar.openbioinformatics.org/en/latest/user-guide/download/, accessed on 12 June 2023) software revealed that 54.1% of the Indels were located in intergenic regions, and 37% were distributed in intronic regions, reflecting the primary distribution patterns in the genome structure.

### 3.2. Population Structure Analysis

PCA revealed that the first two principal components partially explained the genetic variation observed between the groups. Despite some overlap in the PCA distributions of racehorses and non-racehorses, a general trend of separation was observed, suggesting differences in genetic structure between the two groups (Figure 1D).

### 3.3. Genome-Wide Selective Signature Detection

**Figure 2:**
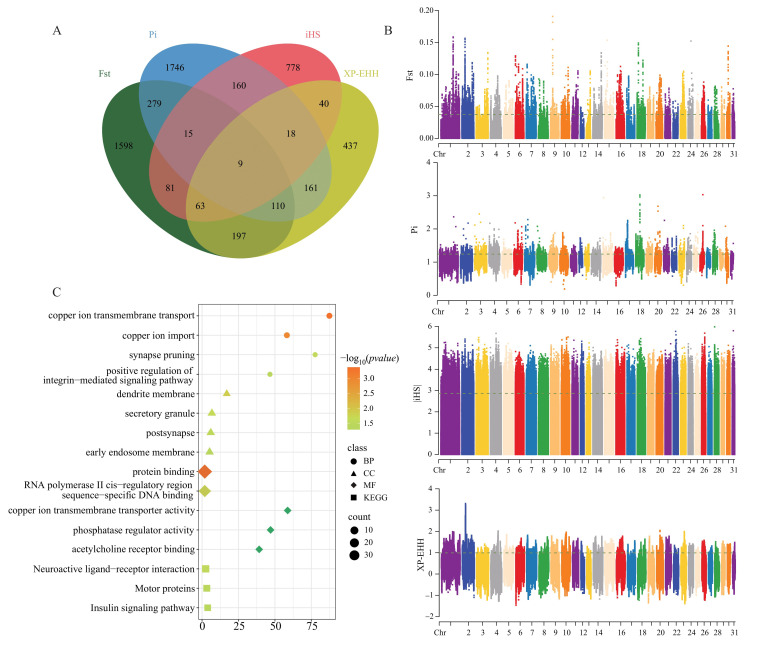
Multiple selection sweep analysis: (**A**) Venn diagram showing the SNPs overlap among Fst, π ratio, his, and XP-EHH. (**B**) Calculation of Fst, π-Ratio, iHS, and XP-EHH values for all autosomes of racing horses using a 100 Kb sliding window with a 10 Kb step size. The green horizontal line indicates the threshold for extracting the top 5% of values for racing horses. (**C**) GO and KEGG pathway enrichment analysis based on SNP candidate genes. The top 5 enriched terms, sorted by corrected *p* < 0.05, are shown below.

Based on the SNP dataset, selection signals associated with traits influencing racing performance were analyzed using four approaches: Fst, π ratio, iHS, and XP-EHH. After filtering for the top 5% overlapping windows, a set of candidate genes were identified (Figure 2B). Specifically, Fst and π ratio analyses identified 11,294 regions (Fst = 0.037, π ratio = 1.24), involving 2352 and 2498 genes, respectively. The iHS and XP-EHH methods, after normalization, identified 1139 regions (|iHS| = 2.86, XP-EHH = 0.99), involving 1164 and 1035 genes, respectively. Using Venn diagram analysis, genes identified shared by at least three of the four methods as candidate genes, resulting in a final set of 215 candidate genes (Figure 2A) (Supplementary Table S4).

**Figure 3:**
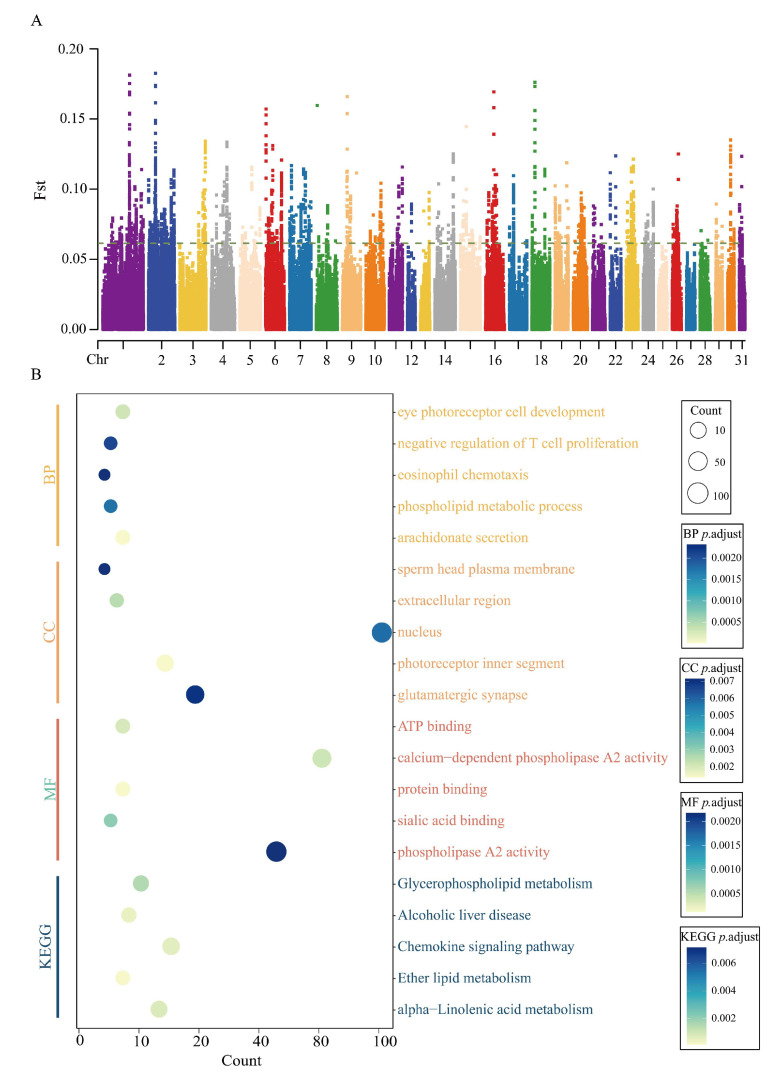
Candidate gene function enrichment analyses: (**A**) Manhattan plot of genome-wide putative selection regions between racing and non-racing horses (Indels dataset). The green horizontal line represents the top 1% of Fst values. (**B**) GO and KEGG pathway enrichment analysis based on Indels candidate genes.

For the analysis of the Indels dataset, the Fst method was applied to identify the top 1% of Indels loci, resulting in 2256 candidate regions (Fst = 0.06) encompassing 661 genes (Figure 3A). The 215 candidate genes derived from the SNP analysis were further integrated with the 661 candidate genes from the Indels analysis (Supplementary Table S5), yielding 84 overlapping genes. These findings suggest that certain genes may play pivotal roles in the selection process underlying racing performance traits, as identified across two independent datasets and analytical methods.

### 3.4. Candidate Gene Enrichment Analysis

Functional enrichment analysis of candidate genes identified from the SNP and Indels datasets was performed using GO and KEGG to elucidate their potential biological functions. Multiple categories were statistically significant (*p* < 0.05). GO enrichment analysis of SNP-derived candidate genes revealed significant enrichment in four biological processes, four cellular components, and six molecular functions. KEGG enrichment identified four critical pathways (Figure 2C). The GO terms were predominantly associated with ion transport, synaptic function, signal transduction, and regulation (Supplementary Table S6), including copper ion transmembrane transport (GO:0035434), dendrite membrane (GO:0032590), protein binding (GO:0005515), and acetylcholine receptor binding (GO:0033130). KEGG pathways were primarily linked to Neuroactive ligand-receptor interaction (ecb04080), Motor proteins (ecb04814), Insulin signaling pathway (ecb04910), and Chronic myeloid leukemia (ecb05220). These pathways influence athletic performance by affecting neural signaling, muscle movement mechanisms, metabolic regulation, and cellular adaptation (Supplementary Table S7).

GO and KEGG enrichment analyses of Indels-derived candidate genes were conducted to investigate the functional impact of mutations in racing and non-racing populations. Approximately 60% of the candidate genes were successfully mapped to GO terms, with the majority enriched in biological processes (Figure 3B). Specifically, 26 biological processes, 13 cellular components, and 14 molecular functions were identified (*p* < 0.05). GO enrichment highlighted functions related to immunity, energy metabolism, and neural regulation (Supplementary Table S8), including ATP binding (GO:0005524), Actin binding (GO:0003779), and Lipid catabolic process (GO:0016042). These functions collectively provide the energy required for physical activity, enhancing endurance and exercise efficiency. KEGG enrichment analysis (Supplementary Table S9) identified 40 key pathways, predominantly related to disease and immune regulation. Pathways closely associated with athletic performance were primarily focused on Motor proteins (ecb04814), the Cytoskeleton in muscle cells (ecb04820), the FoxO signaling pathway (ecb04068), and Fat digestion and absorption (ecb04975). These pathways play crucial roles in energy production, vascular function, and tissue maintenance, all of which are essential for sustaining physical activity and enhancing athletic performance.

**Figure 4:**
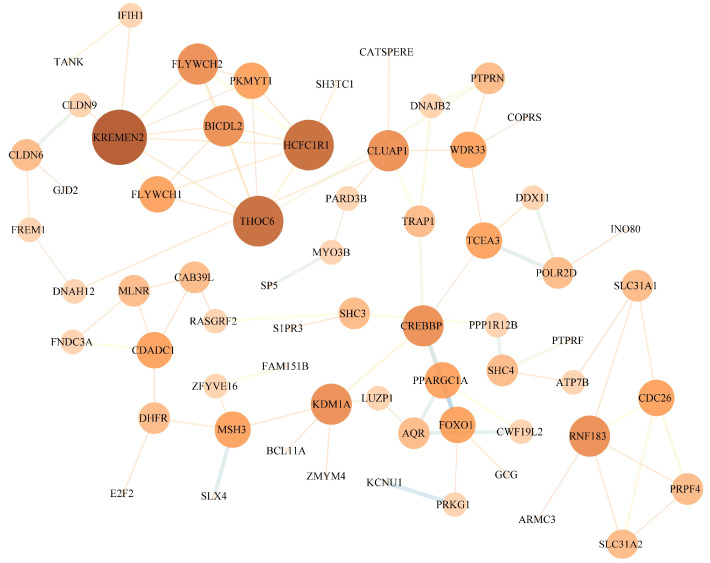
Functional interaction network of candidate genes.

The PPI network suggests that there may be cooperative regulatory functions among these genes (Figure 4). Notably, genes such as *FOXO1*, *PPARGC1A*, and *PRKG1* are located in the network, and these genes play potential key roles in biological processes related to energy metabolism, oxidative stress response, and muscle function. This network provides a preliminary interaction-based perspective on the molecular mechanisms underlying athletic performance in Grassland-Thoroughbreds.

## 4. Discussion

Recent advancements in high-throughput sequencing technologies have enabled the identification of numerous functional genes associated with economically important traits in livestock through selection signal analysis, offering valuable genetic resources for molecular breeding. This study represents the first genomic analysis of locally bred racing breeds, achieving a sequencing coverage of 99.19%, which ensures the accuracy of data analysis. The average sequencing depth reached 25.63, further enhancing the reliability of variant annotation, mutation site identification, and genome structure research. The calculated TS/TV ratio was 2.06, comparable to the ratios observed in human and cattle re-sequencing studies, which were 2.1 and 2.2, respectively. This underscores the high data quality and conserved genome structure, providing a reliable foundation for detecting selection signals [36,37]. The findings of this study not only provide insights for predicting traits related to athletic performance but also establish a scientific basis for breed improvement. These results support the selection and breeding of individuals with superior athletic performance and provide robust data support for the genomic analysis of Grassland-Thoroughbred.

Artificial selection induces significant genetic differentiation in specific genomic regions, influencing racehorse performance. However, racing performance is influenced not only by genetic factors but also by environmental conditions and complex gene interactions [38]. Despite the complexity of this process, the detection of selection signals effectively identifies genes or regulatory genomic elements associated with evolutionary adaptation and essential physiological traits [39,40,41]. Skeletal muscle, a highly plastic tissue, responds to exercise and training stimuli by enhancing athletic performance through adjustments in muscle mass, changes in muscle fiber type composition, and optimization of mitochondrial function. This study identified several key muscle function-related genes through selection analysis. KEGG enrichment highlighted two athletic-performance-associated genes within the insulin signaling pathway: *PPARGC1A* and *FOXO1*. Among these, *PPARGC1A* is a widely recognized gene pivotal to equine athletic performance [42]. *PPARGC1A* encodes PGC-1α in skeletal muscle, which regulates glucose transport during exercise and plays an essential role in promoting mitochondrial biogenesis and adaptation to aerobic training [43,44]. When intense exercise increases energy demands, PGC-1α is activated and collaborates with other transcription factors to regulate the expression of genes involved in insulin signaling, angiogenesis, lipid metabolism, and carbohydrate metabolism [45,46]. Studies indicate that during the recovery phase following exercise, *PPARGC1A* expression and PGC-1α protein levels increase significantly, potentially promoting mitochondrial biogenesis [47,48,49]. Additionally, experiments have demonstrated that in untrained muscle, *PPARGC1A* expression significantly increases following single-leg knee-extension exercises, likely regulated by systemic factors via its canonical promoter. In contrast, trained muscle exhibits a more sustained expression of *PPARGC1A* [49,50,51]. The Gly482Ser polymorphism is regarded as a functional variant of *PPARGC1A*, potentially influencing the differentiation between power- and endurance-oriented athletes. Compared with carriers of the Gly482 allele, individuals with the Ser482 allele generally exhibit lower low-density lipoprotein levels and higher cholesterol levels [52]. The Ser allele frequency is lower among elite endurance and power athletes [53,54]. However, other studies suggest that the Ser allele might benefit power-based sports performance [55]. Regardless of the type of exercise, the Gly allele is generally considered advantageous for endurance sports [56,57]. These findings provide valuable insights into equine sports genetics, suggesting that *PPARGC1A* polymorphisms may serve as targets for optimizing equine athletic performance through gene editing or selective breeding. *PPARGC1A* may enhance the aerobic metabolic capacity of muscle cells in Grassland-Thoroughbreds by promoting mitochondrial biogenesis, enabling them to produce and utilize energy more efficiently during prolonged high-intensity exercise. This knowledge lays a scientific foundation for improving racehorse breeding and enhancing athletic outcomes. *FOXO1* (Forkhead box O1) is a conserved transcription factor across species. Research has demonstrated that *FOXO1* synergizes with PGC-1α, playing a critical role in insulin-mediated glucose and lipid metabolism, while also regulating mitochondrial oxidative stress, autophagy, and overall metabolism [58,59,60,61,62]. Exercise induces an increase in *FOXO1* phosphorylation, enhancing its activity and potentially triggering cardiac hypertrophy, a process demonstrated to exert protective effects on the heart [63,64]. Moreover, *FOXO1* acts as a metabolic switch, promoting a shift towards lipid metabolism [65]. Studies indicate that the mRNA levels of *FOXO1* in muscle are significantly higher in athletes compared with sedentary individuals, highlighting its importance in energy adaptation during physical activity [66]. *FOXO1* is therefore a critical component of the adaptive metabolic response to exercise [63]. Consistently, this study revealed that *PPARGC1A* and *FOXO1* exhibited similar expression patterns in the skeletal muscle of Thoroughbreds, further supporting their roles in energy metabolism and exercise adaptation [67]. *FOXO1* may help Grassland-Thoroughbreds achieve dynamic energy metabolic balance during exercise by regulating glucose and lipid metabolism throughout racing activities. Additionally, the *SGCD* gene encodes δ-sarcoglycan, a protein widely expressed in cardiac and skeletal muscles. It is a vital component of the dystrophin-associated glycoprotein complex [68]. Loss of *SGCD* function leads to muscle dysfunction and may progress to cardiomyopathy characterized by localized fibrosis [69]. δ-sarcoglycan deficiency, caused by mutations in the *SGCD* gene, results in muscle cell damage and degeneration, impairing regenerative capacity and compromising the structural integrity of muscle fiber types [70]. Studies in Drosophila models have demonstrated that *SGCD* deficiency causes muscle tearing and reduced climbing ability [71]. In mice, the absence of δ-sarcoglycan leads to mitochondrial swelling, disrupted cristae structure, loss of membrane integrity, and diminished Ca2+ retention capacity. These alterations are accompanied by reduced oxidative capacity and significantly decreased exercise endurance [72]. *SGCD* may play an important role in maintaining the structural integrity of skeletal and cardiac muscle cells in Grassland-Thoroughbreds and function to prevent muscle damage and degeneration during exercise, thereby ensuring efficient muscle contraction.

The athletic performance of horses is not only constrained by physiological factors but also by psychological and motivational aspects. Genes involved in neurobiological functions frequently appear in transcriptomic and genomic studies investigating equine performance [39]. Among these, the *FOXP2* (Forkhead Box P Subfamily 2) is a highly conserved transcription factor in vertebrates that regulates the development of certain brain functions [73]. Mouse studies have demonstrated that reduced levels of *FOXP2* lead to impaired synaptic plasticity and diminished learning ability, accompanied by abnormalities in motor coordination and sensory functions. Conversely, restoring *FOXP2* expression improves motor coordination and sensory skills [74,75]. *FOXP2* may enhance the neural circuits in the brain related to motor control and sensory processing, thereby improving the coordination and agility of Grassland-Thoroughbreds, which is crucial for precise movements and responses during high-speed racing. Similarly, *PRKG1* (cGMP-dependent protein kinase 1), which is widely expressed in the nervous system, is considered fundamental to neuroplasticity and learning ability [76]. Under high-intensity interval exercise, *PRKG1* expression significantly increases, while the expression of PDE5 (phosphodiesterase 5) decreases. PDE5 phosphorylation activates its enzymatic activity, enhancing its affinity for cGMP within the regulatory domain and reducing intracellular cGMP levels [77]. These findings suggest that neurobiology-related genes, such as *FOXP2* and *PRKG1*, are essential for neural function and behavioral regulation, with potential applications in optimizing equine athletic performance and training adaptability.

Physical exercise significantly elevates the energy demand of skeletal muscle, inducing mitochondrial stress and triggering adaptive responses—hallmarks of exercise training [78]. *SLC25A15* (solute carrier family 25 member 15), an essential member of the mitochondrial carrier family, functions as a mitochondrial ornithine carrier and is integral to arginine synthesis. Studies have demonstrated that during training in male mice, the expression level of *SLC25A15* consistently increases in subcutaneous fat [79]. Arginine can activate AMPK to promote lipolysis, thereby enhancing metabolic efficiency [80]. Furthermore, *SLC25A15* and ornithine contribute to brain energy metabolism homeostasis, cellular ATP transport, and inflammation regulation [81,82]. *CKMT2* (mitochondrial creatine kinase), located in the mitochondrial intermembrane space, catalyzes the rapid hydrolysis of ATP to ADP, facilitating ATP synthesis and supporting mitochondrial respiratory chain activity [83]. Exercise training significantly increases *CKMT2* expression in mice, improving maximum respiration rate and ADP utilization efficiency. This indicates that training-induced mitochondrial functional adaptation in skeletal muscle depends on the intensity of the metabolic load [84]. *TRAP1* (TNF receptor-associated protein 1), a mitochondrial molecular chaperone, regulates the conformation, activity, and stability, thereby modulating cellular metabolism and signaling pathways [85]. *TRAP1* directly interacts with components of the mitochondrial respiratory chain, and its expression level is closely linked to cellular respiratory capacity and glycolysis [86]. In studies related to soccer, *TRAP1* was found to participate in autophagy–lysosome systems and proteasome-mediated protein degradation mechanisms, correlating closely with improved cardiorespiratory function [87,88]. These genes may play important roles in energy supply, endurance, and recovery capacity during competitions in Grassland-Thoroughbreds.

Indels, a type of biallelic genomic variation, are small-scale and widely distributed across the genome, making them a common form of natural genetic variation. As the second most prevalent genomic variation following SNPs [89], Indels are widely employed in human identity verification due to their relatively low and stable mutation rate, which provides an advantage over SNPs and short tandem repeats [90,91]. In this study, both SNPs and Indels were utilized for Fst analysis, revealing largely consistent selective feature regions identified by the two markers. This consistency aligns with findings in other species; for instance, overlapping selective signals between SNPs and Indels have been reported in humans and chickens [92,93]. In equine studies, previous research has linked Indels to various traits, including optimal racing distances, muscle mass [94,95], and coat color [96]. In this research, Indels were also employed to identify selective features, demonstrating that regions surrounding Indels were more frequently subject to recent selective sweeps. Additionally, key genes including *FOXO1*, *SLC25A15*, *CKMT2*, *FOXP2*, and *TRAP1* were found to be influenced by both Indels and SNPs. These genes are enriched in functional pathways such as the FoxO signaling pathway, nuclear function-related pathways, and ATP-binding pathways, which collectively contribute to metabolic regulation, antioxidation, autophagy, muscle adaptation, and energy supply mechanisms. These pathways play critical roles in optimizing athletic performance, particularly in endurance, explosiveness, and recovery [97,98]. The findings underscore the utility of Indel variants as effective tools for analyzing selective traits, while highlighting the significant biological functions of genes influenced by both Indels and SNPs in athletic performance. Nevertheless, further research is required to elucidate the precise mechanisms of Indels and their potential value in selection analysis. Such research is particularly important for validating their consistency across diverse species and environmental contexts. Expanding knowledge in this area could broaden the applications of Indels in genetic breeding and genomic studies.

This study systematically screened candidate genes related to athletic performance in the Grassland-Thoroughbred population, such as *PPARGC1A*, *FOXO1*, and *SGCD*, through whole-genome resequencing, and revealed their potential roles in muscle function. Nevertheless, due to the lack of measurements of key performance indicators and direct validation of gene functions, the genotype–phenotype association analysis in this study still has certain limitations. The relatively small sample size may affect the statistical power of the results and the generalizability of the conclusions. Future studies should increase the sample size to enhance the representativeness and reliability of the research. Secondly, although efforts were made to exclude close relatives through population structure analysis, potential population stratification may still exist, which could introduce bias into the genetic association analysis. Future work needs to collect more athletic performance phenotype data and further explore the genetic basis underlying phenotypic differences in horses. These factors provide opportunities for further exploration in future research. This study provides preliminary genomic resources and candidate gene targets for breed improvement, laying the foundation for future gene function validation, phenotype association studies, and the development of breeding strategies.

## 5. Conclusions

This study conducted a comprehensive analysis of genomic variation in the Grassland-Thoroughbred using whole-genome sequencing (WGS) data and provides preliminary evidence for how these variations may impact racing performance. By employing four different selection signal detection methods, the study identified genetic variations associated with athletic performance, which may contribute to the evolution of adaptive traits crucial for success in horse racing. This study highlighted key candidate genes such as *PPARGC1A*, *FOXO1*, *SGCD*, *FOXP2*, *PRKG1*, *SLC25A15*, *CKMT2*, and *TRAP1*, which play critical roles in muscle function, metabolism, sensory regulation, and neurobiology. While these genes show potential in shaping the racing phenotype, further functional validation experiments are needed to confirm their specific roles in athletic performance. The results provide valuable initial insights for genomic improvement of racing horse populations and lay the foundation for future exploration of the molecular mechanisms underlying equine athletic performance.

## Data Availability

Sequence data that support the findings of this study have been deposited in the National Center for Biotechnology Information with the primary accession code PRJNA11630453 and PRJNA1063377.

## Supplementary Materials

The following supporting information can be downloaded at https://www.mdpi.com/article/10.3390/ani15152323/s1, Supplementary Table S1: The racing records of the Grassland-Thoroughbreds; Supplementary Table S2: The statistical table of sequencing results for each sample; Supplementary Table S3: ANNOVAR annotation results; Supplementary Table S4: SNP candidate genes based on four methods; Supplementary Table S5: Indels candidate genes based on the Fst method; Supplementary Table S6: GO functional enrichment of SNP candidate genes; Supplementary Table S7: KEGG functional enrichment of SNP candidate genes; Supplementary Table S8: GO functional enrichment of Indels candidate genes; Supplementary Table S9: KEGG functional enrichment of Indels candidate genes, Supplementary Figure S1: Breeding route and phylogenetic relationship heatmap of the Grassland-Thoroughbreds.
