# Supplementary material for: Whole-Genome Resequencing Analysis of Athletic Traits in Grassland-Thoroughbred
Source: Animals (Basel). 2025 Aug 7;15(15):2323. doi: 10.3390/ani15152323 (PMC12346297; doi:10.3390/ani15152323)

Author 1

Name: Wenqi Ding

Affiliation: Inner Mongolia Agricultural University

Email: dingwenqi0331@gmail.com

Education Background:		
University name: Inner Mongolia Agricultural University	College name: College of Animal Science	Educational level: Master

Signature:

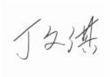

Author 2

Name: Wendian Gong

Affiliation: Inner Mongolia Agricultural University

Email：gongwendian1996@outlook.com

Education Background:		
University name: Inner Mongolia Agricultural University	College name: College of Animal Science	Educational level: Doctor

Signature:

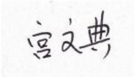

Author 3

Name: Tugeqin Bou

Affiliation: Inner Mongolia Agricultural University

Email: tvgqin@gmail.com

Signature:

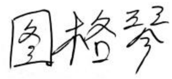

Author 4

Name: Lin Shi

Affiliation: Inner Mongolia Agricultural University

Email: 19832607527@163.com

Education Background：		
University name: Inner Mongolia Agricultural University	College name: College of Animal Science	Educational level：Bachelor

Signature:

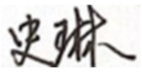

Author 5

Name: Yanan Lin

Affiliation: Inner Mongolia Agricultural University

Email: linyanan@emails.imau.edu.cn

Education Background：		
University name: Inner Mongolia Agricultural University	College name: College of Animal Science	Educational level: Doctor

Signature: .

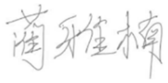

Author 6

Name: Xiaoyuan Shi

Affiliation: Inner Mongolia Agricultural University

Email: xiaoyuans2021@163.com

Education Background：		
University name: Inner Mongolia University	College name: College of Life Science	Educational level：Bachelor

Signature:

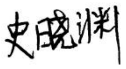

Author 7

Name: Zheng Li

Affiliation: Inner Mongolia Agricultural University

Email: lzheng0511@sina.com

Education Background:		
University name: Inner Mongolia University	College name:　College of Life Science	Educational level：Bachelor

Signature:

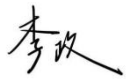

Author 8

Name: Huize Wu

Affiliation: Inner Mongolia Agricultural University

Email: whz020419@163.com

Education Background:		
University name: Inner Mongolia University	College name:　College of Life Science	Educational level：Bachelor

Signature:

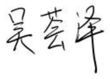

Author 9

Name: Manglai DUGARJAVIIN

Affiliation: Inner Mongolia Agricultural University

Email: dmanglai@163.com

Educational level: Doctor

Signature:

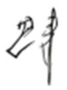

Author 10

Name: Dongyi Bai

Affiliation: Inner Mongolia Agricultural University

Email: baidongyi1983@163.com

Educational level: Doctor

Signature:

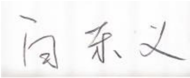

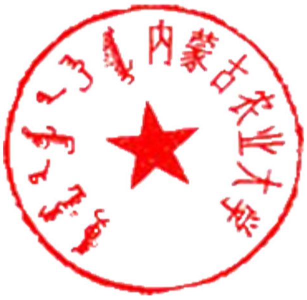

Supplement: Supplementary file 1 [file animals-15-02323-s001.zip › animals-3753935-supplementary/Author's CV.pdf]
