# Supplementary figures and images for: Whole-Genome Resequencing Analysis of Athletic Traits in Grassland-Thoroughbred
Source: Animals (Basel). 2025 Aug 7;15(15):2323. doi: 10.3390/ani15152323 (PMC12346297; doi:10.3390/ani15152323)

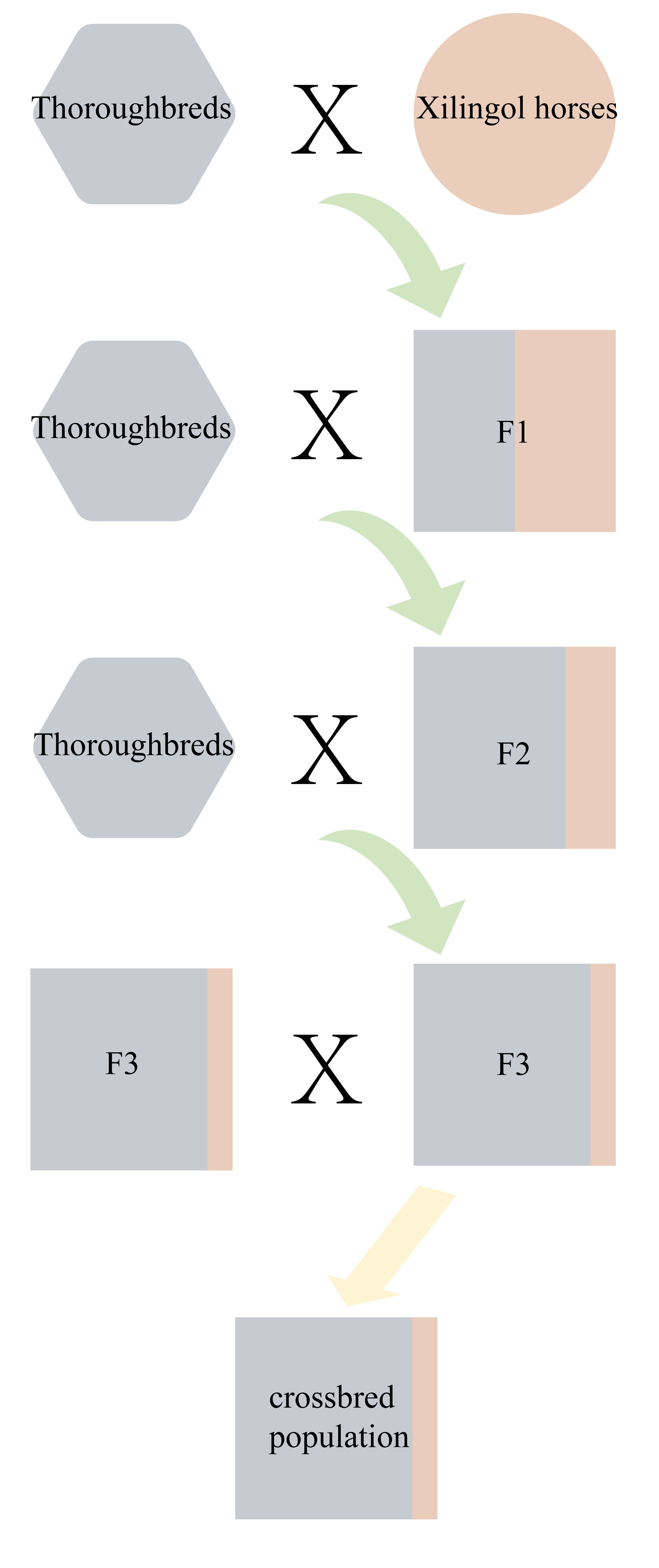

Supplement: Supplementary file 1 [file animals-15-02323-s001.zip › animals-3753935-supplementary/Supplementary Figure S1 Breeding route of the Grassland Thoroughbreds.png]
